# Similarity-Driven Fine-Tuning Methods for Regularization Parameter Optimization in PET Image Reconstruction

**DOI:** 10.3390/s23135783

**Published:** 2023-06-21

**Authors:** Wen Zhu, Soo-Jin Lee

**Affiliations:** Department of Electrical and Electronic Engineering, Pai Chai University, Daejeon 35345, Republic of Korea; 2023601@pcu.ac.kr

**Keywords:** image reconstruction, penalized likelihood methods, regularization parameters, patch similarity, positron emission tomography

## Abstract

We present an adaptive method for fine-tuning hyperparameters in edge-preserving regularization for PET image reconstruction. For edge-preserving regularization, in addition to the smoothing parameter that balances data fidelity and regularization, one or more control parameters are typically incorporated to adjust the sensitivity of edge preservation by modifying the shape of the penalty function. Although there have been efforts to develop automated methods for tuning the hyperparameters in regularized PET reconstruction, the majority of these methods primarily focus on the smoothing parameter. However, it is challenging to obtain high-quality images without appropriately selecting the control parameters that adjust the edge preservation sensitivity. In this work, we propose a method to precisely tune the hyperparameters, which are initially set with a fixed value for the entire image, either manually or using an automated approach. Our core strategy involves adaptively adjusting the control parameter at each pixel, taking into account the degree of patch similarities calculated from the previous iteration within the pixel’s neighborhood that is being updated. This approach allows our new method to integrate with a wide range of existing parameter-tuning techniques for edge-preserving regularization. Experimental results demonstrate that our proposed method effectively enhances the overall reconstruction accuracy across multiple image quality metrics, including peak signal-to-noise ratio, structural similarity, visual information fidelity, mean absolute error, root-mean-square error, and mean percentage error.

## 1. Introduction

Positron emission tomography (PET) is a non-invasive imaging technique that enables the visualization of biochemical processes in the patient body by using a radioactive substance known as a radiotracer [1,2]. The patient undergoing the PET scan is injected with the radiotracer, which travels through the body and gets absorbed by the targeted organ or tissue. Once the radiotracer is injected into the body, it begins to emit positrons. When a positron collides with an electron in the surrounding tissue, it annihilates and produces two gamma rays in opposite directions. These gamma rays are detected by a ring of detectors surrounding the patient. The aim of image reconstruction in this case is to accurately map the distribution of the radioactive material in the patient’s body, which can provide valuable information about various physiological and biochemical processes. However, PET images are often characterized by low spatial resolution and high noise, which can limit their diagnostic accuracy. To address these limitations, various image reconstruction methods have been developed over the last decades, which aim to improve the spatial resolution and signal-to-noise ratio of PET images and reduce the amount of radiation exposure required for accurate imaging [3].

Among the various reconstruction methods, the penalized-likelihood (PL) approach, which is also known as the model-based iterative reconstruction method, has been shown to offer remarkable advantages over the traditional filtered back-projection method by providing improved spatial resolution, reduced imaging noise, and increased detection sensitivity [3,4,5,6]. The PL approach is a statistical method that uses the measured data and a mathematical model of the imaging process to estimate the distribution of radioactivity in the patient’s body, while also applying a penalty function (or a regularizer) that promotes spatial smoothness and noise reduction.

Recently, inspired by the rapid development of artificial intelligence in a variety of research and industrial fields, efforts have been made to improve the quality of medical images using deep learning techniques [7,8,9,10,11,12,13]. For tomographic image reconstruction, deep learning methods have also been applied to the PL reconstruction methods [14,15]. However, the PL methods involve hard-to-find hyperparameters (also known as regularization parameters) that significantly affect the quality of reconstructed images. The selection of appropriate regularization parameters is a challenging task, as it involves balancing the trade-off between noise reduction and preservation of important features in the underlying image. Moreover, the optimal regularization parameters may vary depending on the specific imaging task and the characteristics of the data being reconstructed.

Over the years, several methods for automatic parameter adjustment have been developed [16,17,18,19,20]. The representative early methods include the L-curve [16] and generalized cross-validation (GCV) [17,18] methods. The L-curve method relies on the shape of the L-curve indicating the trade-off between data fidelity and regularization so that the corner point of the L-curve is chosen as the optimal regularization parameter. The GCV method relies on the mean squared error and effective degrees of freedom to determine the optimal parameters. While the L-curve method typically requires multiple reconstructions with different regularization parameters to obtain the L-curve, the GCV method is computationally efficient since it avoids the need for repeated reconstructions with different regularization parameters. It has also been reported that assessing image quality can guide hyperparameter adjustment [19,20].

Recently, deep learning-based hyperparameter-tuning methods have been proposed in the literature [21,22,23]. The method presented in [21] exhibits an intelligent approach by utilizing deep reinforcement learning to determine the direction and magnitude of parameter adjustment in a human-like manner. However, this method learns a hyperparameter tuning strategy based on feedback from intermediate image reconstruction results, which necessitates running multiple iterations of an image reconstruction algorithm before parameter adjustment. This process has the potential to significantly decrease the overall workflow efficiency. In contrast, the methods proposed in [22,23] employ convolutional neural network-based hyperparameter learning frameworks. These frameworks employ a training pair consisting of the sinogram as the input and the desirable hyperparameter as the output. Although these methods generate hyperparameters in a feedforward manner once the network is trained, their applicability is limited to simple quadratic smoothing regularization, rather than edge-preserving non-quadratic regularization.

Here, we note that, for edge-preserving regularization, in addition to the smoothing parameter that balances data fidelity and regularization, one or more control parameters are typically incorporated to adjust the sensitivity of edge preservation by modifying the shape of the penalty function [24]. Without appropriately selecting these control parameters, it is challenging to obtain high-quality images. Unfortunately, the parameter-tuning methods discussed in [16,17,18,19,20,21,22,23] primarily focus on the smoothing parameter. In this work, to enhance the efficacy of existing parameter-tuning methods, we propose a method to precisely tune the hyperparameters, which are initially set with a fixed value for the entire image, either manually or using an automated approach. The fundamental strategy involves adjusting the initial value of the control parameter at each pixel, either increasing or decreasing it, based on the degree of the patch similarities calculated from the previous iteration within the pixel’s neighborhood that is being updated. This approach allows our new method to integrate with a wide range of existing parameter-tuning techniques from prior research.

Our work is inspired by the well-known non-local means approach [25], which has been widely used for image denoising [25,26,27,28,29] and restoration/reconstruction [30,31,32,33,34] by exploiting the measure of similarity between the image patches. The nonlocal means approach is based on the idea that in an image, pixels that are similar to each other tend to have similar values. Therefore, instead of averaging the values of neighboring pixels to obtain an estimate of the value of a particular pixel, the nonlocal means approach takes into account the similarity between the patches centered on each pixel in the image. The weighted average of the patch values is then used to obtain an estimate of the value of the pixel of interest. While our work is inspired by the non-local means approach, our method for fine-tuning the control parameter differs from the non-local means denoising approach. Instead of using the similarity measure between patches to calculate the weighted average for edge-preserving smoothing, our approach applies the similarity measure to calculate the optimal value for the control parameter for each pixel. The experimental results demonstrate that our proposed method enables adaptive selection of the optimal control parameter for each pixel, leading to enhanced image quality in the reconstruction process.

The remainder of this paper is organized as follows: Section 2 first describes the PL approach to PET image reconstruction and illustrates the two representative edge-preserving convex non-quadratic (CNQ) penalty functions, which involve the hyperparameters controlling the sensitivity of edge preservation. The details about our main idea of using the similarity-driven method for hyperparameter tuning are then described. The optimization method for the PL reconstruction algorithm with the CNQ penalty functions is also derived. Section 3 shows our experimental results using both digital and physical phantoms, where our proposed method effectively enhances the overall reconstruction accuracy across multiple image quality metrics. Finally, Section 4 draws a conclusion.

## 2. Methods

### 2.1. Penalized Likelihood Approach

The PL approach to PET image reconstruction is to seek the estimate $\hat{f}$ of the underlying source image f from the emission measurement g by using the following minimization:

(1) $$\hat{f}=\underset{f}{\arg\min}\left[-L(g|f)+\lambda R(f)\right]$$

where L(g|f) is the log-likelihood term represented by the log of a Poisson distribution, R(f) is the regularization term to penalize the image roughness, λ is the smoothing parameter that controls the balance between the two terms. The regularization term is usually defined in such a way that it penalizes the roughness of the estimate by the intensity difference between neighboring pixels, which is given by

(2) $$R(f)=\sum_{j}\sum_{j^{\prime }\in N_{j}}\varphi (f_{j}-f_{j^{\prime }}),$$

where φ(⋅) is the penalty function, $f_{j}$ is the *j*-th pixel in an image, $f_{j^{\prime }}$ is the neighbor of $f_{j}$, and $N_{j}$ is the neighborhood system of the pixel $f_{j}$.

**Figure 1:**
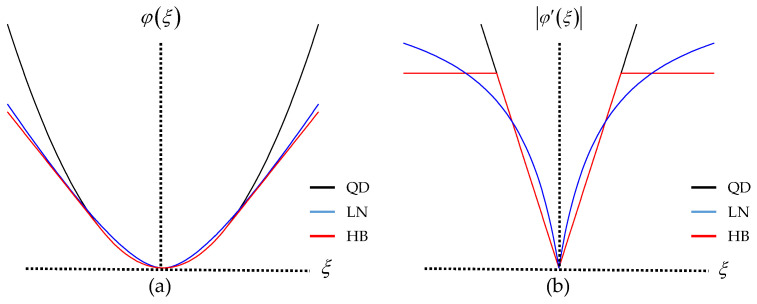
Three representative penalty functions: (**a**) typical shapes of the three (QD, LN, and HB) penalty functions; (**b**) first-order derivatives of the three penalty functions indicating the strength of smoothing.

In this work, among many different convex non-quadratic (CNQ) penalty functions, we consider the following two most popular CNQ functions proposed by Lange [35] (denoted as LN hereafter) and Huber [36] (denoted as HB hereafter):

(3) $$\varphi_{LN}(\xi )=\delta^{2}\left[\left|\frac{\xi }{\delta }\right|-\log\left(1+\left|\frac{\xi }{\delta }\right|\right)\right],$$

(4) $$\varphi_{HB}(\xi )=\left\{\begin{array}{c} \xi^{2},\\ 2\sigma \left|\xi \right|-\sigma^{2}, \end{array}\begin{array}{c} \left|\xi \right|\leq \sigma \\ \left|\xi \right|>\sigma \end{array}\right.,$$

where *δ* and *σ* are the positive hyperparameters that control the sensitivity of edge preservation by modifying the shape of the penalty functions $\varphi_{LN}(\cdot )$ and $\varphi_{HB}(\cdot )$, respectively. The typical shapes of the LN and HB penalty functions are shown in Figure 1, where they are compared with the quadratic (QD) penalty function $\varphi_{QD}(\xi )=\xi^{2}$.

Based on the observation in Figure 1a, it can be seen that the CNQ penalty functions exhibit lower penalization than the QD penalty for significant intensity differences between the adjacent pixels. This characteristic enables the CNQ penalties to effectively preserve edges. The first-order derivative of the penalty function in Figure 1b indicates the strength of smoothing. Comparing it to the QD penalty function, which shows a linear increase in the magnitude of the derivative with increasing intensity difference, the LN penalty demonstrates a slower increase beyond a large value of the intensity difference. Furthermore, the HB penalty remains constant once the intensity difference reaches a large value. Therefore, both the LN and HB penalty functions satisfy the necessary condition for a CNQ penalty function to preserve edges, which is summarized as $\underset{\xi \to \infty }{\lim}\left|\varphi^{\prime }(\xi )\right|=K,$ where $\varphi^{\prime }(\xi )$ is the first-order derivative of the penalty function and K is a positive constant [37]. For a given intensity difference between the adjacent pixels, as the hyperparameter *δ* (or *σ*) decreases, K also decreases, which results in more edges, and vice versa. To effectively preserve edges while suppressing noise, selecting an appropriate value for the hyperparameter is crucial. In this work, we assume that all hyperparameters (*λ*, *δ*, and *σ*) are preselected for the entire image before the reconstruction process begins. We aim to refine the value of *δ* (or *σ*) for each pixel during the reconstruction process by using the patch similarities within the neighborhood of a pixel to be updated. This approach enables us to fine-tune the hyperparameter value on a per-pixel basis, optimizing edge preservation in the reconstructed image.

### 2.2. Similarity-Driven Hyperparameter Tuning

In this work, inspired by the well-known non-local mean (NLM) approach [25], which has shown great potential in removing noise while preserving image details such as edges and textures by exploiting the redundancy and self-similarity of the image structure, we propose a new method of fine-tuning the hyperparameter *δ* (or *σ*) by using the self-similarity of the underlying image structure. The NLM approach is based on the idea that in an image, pixels that are similar to each other tend to have similar values. Therefore, instead of averaging the values of neighboring pixels to obtain an estimate of the value of a particular pixel, the NLM approach takes into account the similarity between the patches centered on each pixel in the image and computes a weighted average of patches centered around each pixel in the image.

**Figure 2:**
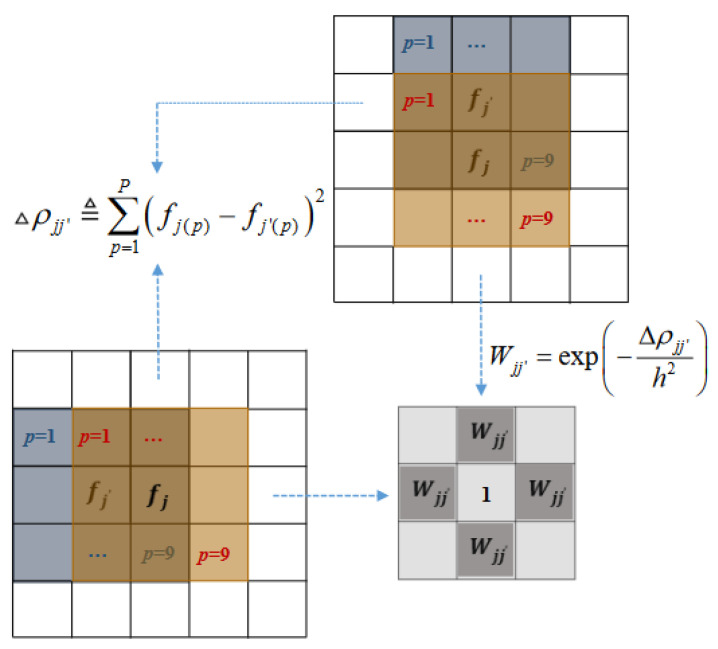
Calculating the patch similarity matrix $W_{j}$ using a 3 × 3 patch window. The similarity matrix $W_{j}$ consists of the four elements in the neighbors and one $\left(W_{jj}=1\right)$ in the center.

In the NLM approach, the similarity between the two patches is defined by [25]

(5) $$W_{jj^{\prime }}=\exp\left(-\frac{△\rho_{jj^{\prime }}}{h^{2}}\right),$$

where $△\rho_{jj^{\prime }}$ is the patch difference and *h* is a positive parameter. The patch difference $△\rho_{jj^{\prime }}$ is defined as

(6) $$△\rho_{jj^{\prime }}≜{\left\|\rho (N_{j})-\rho (N_{j^{\prime }})\right\|}^{2}=\sum_{p=1}^{P}{\left(f_{j(p)}-f_{j^{\prime }(p)}\right)}^{2},$$

where $\rho (N_{j})$ and $\rho (N_{j^{\prime }})$ are the patches centered at the pixel *j* and *j′*, respectively, *P* is the total number of pixels in a patch, and $f_{j(p)}$ and $f_{j^{\prime }(p)}$ are the *p*-th pixels in the patches $\rho (N_{j})$ and $\rho (N_{j^{\prime }})$, respectively. For a 3 × 3 patch window, $△\rho_{jj^{\prime }}$ defined in (6) can be calculated by visiting each of the 9 pixels (p=1,2,…,9). Figure 2 shows how the similarity matrix $W_{j}$ is calculated when the neighborhood system $N_{j}$ consists of four neighbors (north, south, east, and west) and one $\left(W_{jj}=1\right)$ in the center.

Note that the NLM approach in image denoising uses the similarity between the two patches defined by (5) for weighted smoothing, which can be expressed as

(7) $$R(f)=\sum_{j}\sum_{j^{\prime }\in N_{j}}\omega_{jj^{\prime }}\varphi (f_{j}-f_{j^{\prime }}),\;\mathrm{where}\;\omega_{jj^{\prime }}=W_{jj^{\prime }}/\sum_{j\prime \in N_{j}}W_{jj^{\prime }}.$$

In contrast, our method uses the similarity in (5) to adjust the initially tuned value of *δ* (or *σ*). The basic strategy to refine the initially tuned parameter $\delta =\delta^{0}$ is that the value of *δ* at a pixel may be increased or decreased depending on the degree of the patch similarity. To incorporate the patch similarity $W_{jj^{\prime }}$ into the adjustment of *δ*, we use the following formula:

(8) $$\delta_{jj^{\prime }}=\delta^{0}\left[1+\left(W_{jj^{\prime }}+\alpha_{j}w\right)\right],$$

where $\delta_{jj^{\prime }}$ is the fine-tuned value of *δ* using the patch similarity between the two patches centered at the pixels *j* and *j′*, *w* is the mean of $W_{jj^{\prime }}$ evaluated for all pixels in the estimated image obtained from the previous iteration of the PL reconstruction process, and $\alpha_{j}$ is in [−1,1] which is also determined from the previous iteration by measuring the degree of roughness within the neighborhood of the pixel *j.* (The value of $\alpha_{j}$ approaches −1 when the pixel roughness is very low, whereas it approaches 1 when the roughness is very high.) In (8), a negative value of $\alpha_{j}$ decreases $\delta_{jj^{\prime }}$, whereas a positive value of $\alpha_{j}$ increases $\delta_{jj^{\prime }}$. In an extreme case, where $\alpha_{j}=-1$ due to the irregular edges with relatively low similarities, the value of $\delta_{jj^{\prime }}$ can be smaller than $\delta^{0}$. On the other hand, when $\alpha_{j}=-1$ due to the regular edges with high similarities, $\delta_{jj^{\prime }}$ can be close to $\delta^{0}.$ When $\alpha_{j}=1$ in a flat region, $\delta_{jj^{\prime }}$ is larger than $\delta^{0}$. In summary, $\delta_{jj^{\prime }}$ adaptively varies around $\delta^{0}$ depending on the patch similarities in the neighborhood of the pixel *j.*

**Figure 3:**
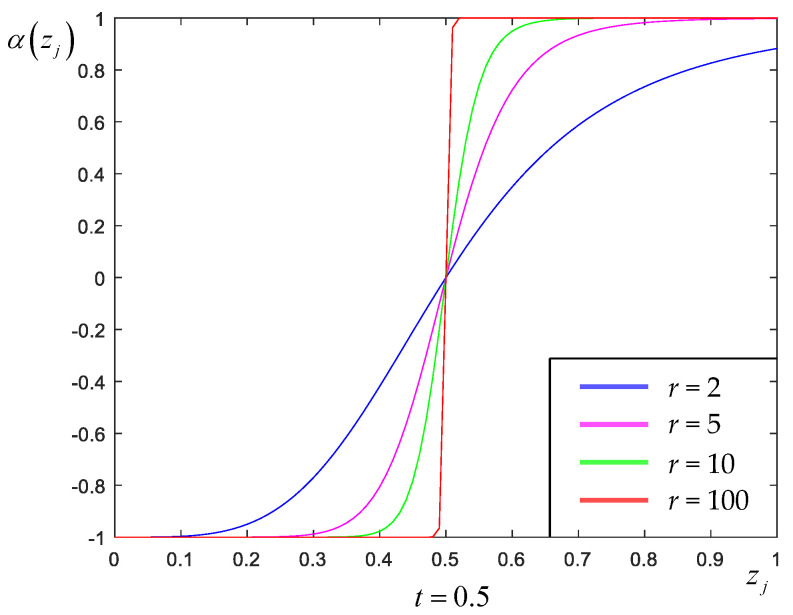
Plot of the modified *r*-th order Butterworth polynomial $\alpha \left(z_{j}\right)$ with several different values of *r*.

To avoid a sudden change of the sign of $\delta_{jj^{\prime }}$, we define $\alpha_{j}$ using the following modified Butterworth polynomial:

(9) $$\alpha \left(z_{j}\right)=\frac{2}{1+{\left(t/z_{j}\right)}^{2r}}-1,\;\alpha \in \left[-1,1\right],$$

where *t* is the turning point of the *r*-th order polynomial, $z_{j}$ is the *j*-th pixel in the image **z** standing for the pixel-wise roughness in the estimated image obtained from the previous iteration of the reconstruction process. Various pixel-wise roughness measures may be used for **z**. In this work, we compare the three different roughness measures: gradient (GR), standard deviation (SD), and mean of patch similarity (PS). The GR of an image is a vector field that represents the magnitude and direction of the change in intensity at each pixel in the image. To measure the pixel-wise roughness only, the magnitude of the GR is used. The pixel-wise SD image is calculated as follows:

(10) $$s_{j}=\sqrt{\frac{1}{L-1}{\sum_{k\in N_{j}}\left[f_{k}-\left(\frac{1}{L}\sum_{j^{\prime }\in N_{j}}f_{j^{\prime }}\right)\right]}^{2}},\;\forall j,,$$

where $s_{j}$ is the *j*-th pixel in the SD image calculated within the 3×3 neighborhood system $N_{j}$ of the pixel $f_{j}$ and *L* = 9 in this case. The mean of patch similarity for the *j*-th pixel is defined by $W_{j}=\sum_{j^{\prime }\in N_{j}}W_{jj^{\prime }}$. Figure 3 shows shapes of $\alpha \left(z_{j}\right)$ in (9) for several different values of *r*. (In our experiments, the value of *t* was set to the mean of **z** and *r* = 0.1*λ*.)

### 2.3. Derivation of PL Reconstruction Algorithm

To derive a PL reconstruction algorithm that employs the similarity-driven fine-tuning method for hyperparameter optimization, we first use an accelerated version of the maximum likelihood (ML) algorithm, namely, the complete data ordered subsets expectation–maximization (COSEM) [38] algorithm and extend it to a PL algorithm to include the regularization term. While the well-known ordered subsets expectation–maximization (OSEM) [39] algorithm accelerates the original expectation maximization (EM) algorithm [40] by subdividing the projection data into several subsets (or blocks) and then progressively processing each subset by performing projection and back-projection operations in each iteration, it is not provably convergent due to the lack of its objective function. On the other hand, the COSEM algorithm is fast and convergent with an objective function.

The COSEM algorithm applies the idea of ordered subsets used in the OSEM algorithm on the “complete data” **C** rather than on the projection data **g**. The complete data **C**, whose elements are denoted as $C_{ij}$, represents the number of coincidence events that originated at the *j*-th pixel in the underlying source and recorded by the *i*-th detector pair so that the following relationship holds: $\sum_{j}C_{ij}=g_{i}$.

The COSEM-ML algorithm can be expanded to the COSEM-PL algorithm by including the regularization term. For our COSEM-PL algorithm, if **C** is fixed to $C=C^{\left(n\right)}$ at the *n*-th iteration in an alternative updating procedure, the overall energy function with the regularizer in (2) can be expressed as:

(11) $$E\left(f;C^{\left(n\right)}\right)=-\sum_{q=1}^{Q}\sum_{i\in S_{q}}\sum_{j}C_{ij}^{\left(n,q\right)}\log f_{j}+\sum_{ij}H_{ij}f_{j}+\lambda R\left(f\right),$$

where $S_{q}$, *q* = 1,…, *Q*, is the *q*-th subset of the detector pairs, and $C_{ij}^{\left(n,q\right)}$ denotes the update of $C_{ij}$ at outer iteration *n* and subset iteration *q*. When the regularization term in (11) takes a CNQ form as described by (3) or (4), it is not possible to obtain a closed-form solution. Therefore, we employ the method of optimization transfer using paraboloidal surrogates [41,42,43] that can efficiently find a global minimum of a convex function by using the following surrogate function for the penalty term [42]:

(12) $$\hat{\phi }(\xi )=\phi (\xi^{n-1})+\phi^{\prime }(\xi^{n-1})(\xi -\xi^{n-1})+\frac{1}{2}\psi (\xi^{n-1}){\left(\xi -\xi^{n-1}\right)}^{2}\geq \phi (\xi ),$$

where the $\varphi^{\prime }\left(\xi \right)$ is the first-order derivative, $\xi^{n-1}$ denotes the value of ξ at the (*n* − 1)-th iteration, and $\psi \left(\xi \right)=\varphi^{\prime }\left(\xi \right)/\xi$. By dropping the terms that are independent of the variable ξ, (12) can be written as

(13) $$\hat{\varphi }\left(\xi \right)=\frac{1}{2}\psi \left(\xi^{n-1}\right)\xi^{2}.$$

To avoid the coupling problem of $f_{j}$ and $f_{j^{\prime }}$ when ξ is substituted with $f_{j}-f_{j\prime }$ in the quadratic term in (13), the regularization term is modified by using the separable paraboloidal surrogate (SPS) function [44,45] as follows:

(14) $$\hat{R}\left(f;f^{n-1}\right)=\sum_{j}\sum_{j^{\prime }\in N_{j}}\psi \left(f_{j}{}^{n-1}-f_{j^{\prime }}{}^{n-1}\right){\left(2f_{j}-f_{j}{}^{n-1}-f_{j^{\prime }}{}^{n-1}\right)}^{2}$$

By replacing the regularization term in (11) with $\hat{R}\left(f_{j};f^{n-1}\right)$, the overall energy function for each $f_{j}$ is expressed as

(15) $$\begin{array}{l} E\left(f_{j};f^{\left(n,q-1\right)},C^{\left(n,q\right)}\right)=-\sum_{i}C_{ij}^{\left(n,q\right)}\log f_{j}+\sum_{i}H_{ij}f_{j}\\ \;\;\;\;\;\;\;+\lambda \sum_{j^{\prime }\in N_{j}}\psi \left(f_{j}{}^{\left(n,q-1\right)}-f_{j^{\prime }}{}^{\left(n,q-1\right)}\right){\left(2f_{j}-f_{j}{}^{\left(n,q-1\right)}-f_{j^{\prime }}{}^{\left(n,q-1\right)}\right)}^{2}, \end{array}$$

where $f_{j}^{\left(n,q\right)}$ denotes the update of $f_{j}$ at outer iteration *n* and subset iteration *q*. Note that after the completion of the subset iteration at the *n*-th iteration, $f^{\left(n,Q\right)}$ is assigned to $f^{\left(n+1\right)}$. By setting the derivative of (15) to zero and solving for the positive root of the quadratic equation, the final update equation is given by

(16) $$f_{j}^{\left(n,q\right)}=\frac{-b+\sqrt{b^{2}-4ac}}{2a},$$

where *a*, *b,* and *c* are given by

$$a=8\lambda \sum_{j^{\prime }\in N_{j}}\psi \left(f_{j}{}^{\left(n,q-1\right)}-f_{j^{\prime }}{}^{\left(n,q-1\right)}\right),\;b=\sum_{i}H_{ij}-4\lambda \sum_{j^{\prime }\in N_{j}}\psi \left(f_{j}{}^{\left(n,q-1\right)}-f_{j^{\prime }}{}^{\left(n,q-1\right)}\right)\left(f_{j}{}^{\left(n,q-1\right)}+f_{j^{\prime }}{}^{\left(n,q-1\right)}\right),\;c=-\sum_{j}C_{ij}{}^{\left(n,q\right)}.$$

In the COSEM-PL algorithm, the **C**-update is the same as the **C**-update in the COSEM-ML algorithm. Therefore, the COSEM-PL algorithm is performed by alternately updating $C_{ij}{}^{\left(n,q\right)}$ and $f_{j}^{\left(n,q\right)}$ at outer iteration *n* and subset iteration *q*.

**Figure 4:**
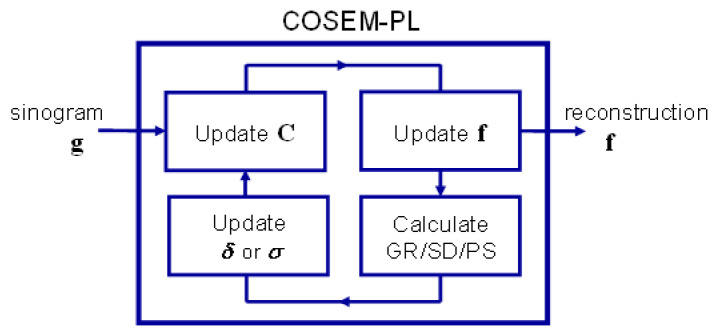
Schematic diagram of the COSEM-PL algorithm with adaptive parameter tuning.

Figure 4 shows the schematic diagram of the COSEM-PL algorithm, where our parameter fine-tuning method is applied. Note that the control parameter *δ* (or *σ*) is updated using one of the three roughness measures (GR, SD, and PS) calculated from the image reconstructed in the previous iteration, and the initial values of the hyperparameters (*λ*, *δ* or *σ*) are preset either manually or using an automated method before the COSEM-PL iteration begins.

## 3. Results

### 3.1. Numerical Studies Using Digital Phantom

**Figure 5:**
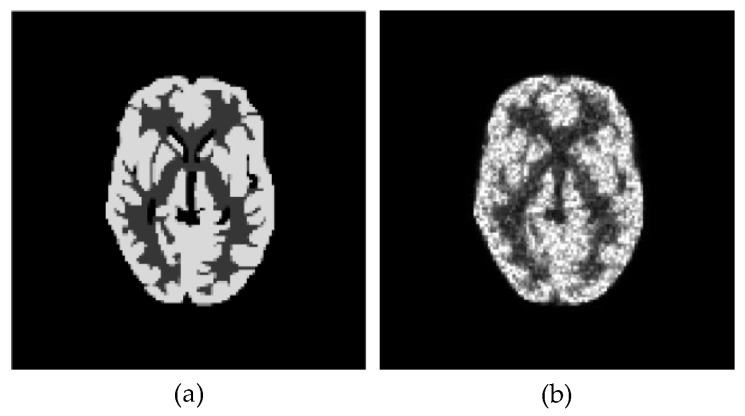
Digital phantom used in simulations and typical 500,000-count noise level for EM-ML reconstruction: (**a**) 128 × 128 digital Hoffman brain phantom; (**b**) EM-ML reconstruction (40 iterations) from noisy projection data with 500,000 photon counts.

To test our idea, we first performed numerical studies using a 128 × 128 digital Hoffman brain phantom slice shown in Figure 5a. The activity ratio of the phantom is 4:1:0 in gray matter, white matter, and cerebrospinal fluid (CSF), respectively. For projection data, we used 128 projection angles over 180° with 128 detector pairs. To generate projection data with noise, we first scaled the phantom so that the total counts of its projection data could be approximately 500,000, and then added independent Poisson noise to the noiseless projection data obtained from the scaled phantom. Figure 5b provides a qualitative representation of the typical noise level observed in the 40th iteration of the EM-ML (or the COSEM-ML with a single subset) reconstruction from a noisy sinogram with approximately 500,000 photon counts.

For PL reconstruction, we compared two different methods: the standard PL method, which uses fixed hyperparameter values for all pixels in the entire image, and the similarity-driven PL (SDPL) method, which employs our proposed method of parameter fine-tuning on a per-pixel basis. To ensure convergence, we used 4 subsets and 80 iterations, which effectively corresponds to 320 iterations for a single subset. To assess the effectiveness of the SDPL algorithm across diverse hyperparameter configurations, we employed two distinct (high and low) levels of initial parameter values for both the smoothing parameter *λ* and the control parameter *δ* (or *σ*). Note that our approach can seamlessly integrate with a wide range of existing parameter-tuning methods, thereby eliminating the need for a specific criterion in selecting the initial parameter values.

**Figure 6:**
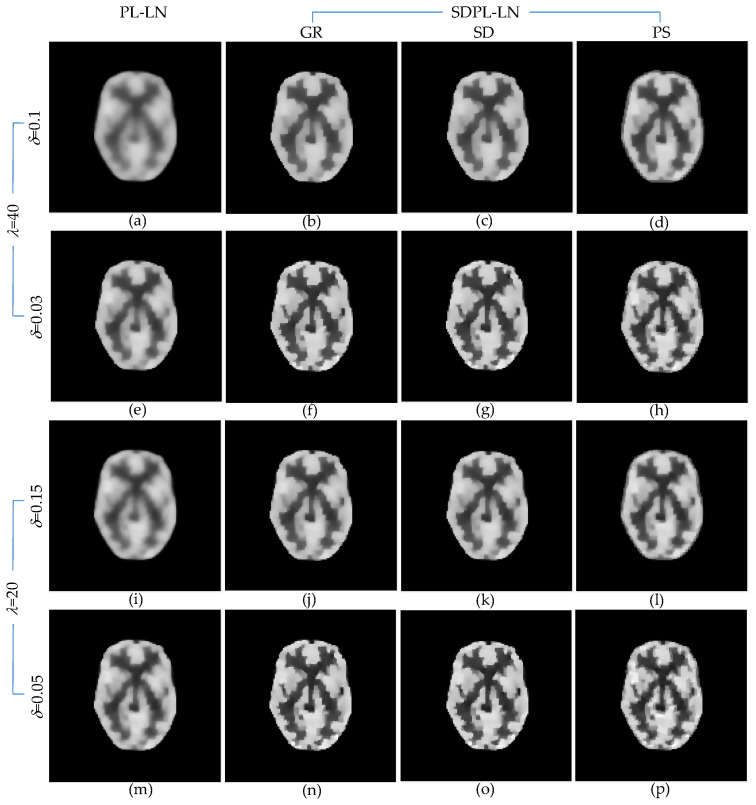
Anecdotal reconstructions using PL-LN and SDPL-LN with two different (high and low) levels of *λ* and two different (high and low) levels of *δ* for each *λ*. (The results in the first column (**a**,**e**,**i**,**m**) are PL-LN reconstructions, whereas the rest of the results are SDPL-LN reconstructions). (**a**–**h**) *λ* = 40 (**i**–**p**) *λ* = 20 (**a**–**d**) *δ* = 0.1; (**e**–**h**) *δ* = 0.03; (**i**–**l**) *δ* = 0.15; (**m**–**p**) *δ* = 0.05.

Figure 6 shows the anecdotal PL and SDPL reconstructions using the LN penalty function. The figure comprises four groups of results, each corresponding to a different parameter setting. Specifically, Figure 6a–d shows the results obtained with high *λ* and high *δ*, Figure 6e–h with high *λ* and low *δ*, Figure 6i–l with low *λ* and high *δ*, and Figure 6m–p with low *λ* and low *δ*. Within each row, the reconstruction methods are displayed from left to right as PL-LN and SDPL-LN (GR, SD, and PS), respectively. A qualitative comparison of the results in Figure 6 clearly reveals that the SDPL method better preserves fine details than the standard PL method.

To elaborate further, when both *λ* and *δ* are excessively large (Figure 6a–d), the PL result in Figure 6a appears over-smoothed, whereas the SDPL results in Figure 6b–d exhibit enhanced detail. By reducing the value of *δ* while keeping *λ* fixed, the SDPL result in Figure 6e becomes sharper than its PL counterpart in Figure 6a. Similarly, the SDPL results in Figure 6f–h, like those in Figure 6b–d, demonstrate superior preservation of fine details compared to the result in Figure 6e. Based on the observations from Figure 6a–h, we tentatively conclude that the SDPL method effectively mitigates the over-smoothing issue of the PL method for relatively high *λ* values. As expected, when the smoothing parameter *λ* is decreased, the results become sharper and exhibit more details. However, even in this case, the SDPL method further enhances reconstruction accuracy by better preserving fine details, as evident in Figure 6i–p. In an extreme case, where the values of both *λ* and *δ* are very small, the results become noisy, a phenomenon that is not specific to the SDPL method but holds true for any regularization method. In conclusion, the SDPL method surpasses the standard PL method in effectively preserving fine details when the hyperparameters are chosen to be sufficiently large, ensuring effective noise suppression.

To evaluate and compare, in an ensemble sense, the quantitative performance of the reconstruction algorithms with the parameter settings used for Figure 6, we generated 50 independent noise realizations of projection data for the phantom shown in Figure 5a.

Table 1 presents a quantitative performance comparison between the PL-LN and SDPL-LN in terms of six different image quality assessments (IQAs): peak signal-to-noise ratio (PSNR); structural similarity (SSIM); visual information fidelity (VIF); mean absolute error (MAE); root-mean-square error (RMSE); and mean percentage error (MPE). All IQA metrics used in this work were evaluated from 50 independent Poisson noise trials. For example, the MPE is defined as

(17) $$MPE=\frac{1}{K}\sum_{k=1}^{K}\sqrt{\sum_{}{\left({\hat{f}}_{j}^{k}-f_{j}\right)}^{2}/\sum_{}f_{j}{}^{2}}\times 100\%,$$

where ${\hat{f}}_{j}^{k}$ is the *j*-th pixel value of the reconstructed image for the *k*-th noise trial, $f_{j}$ is the *j*-th pixel value of the noiseless phantom, and K=50 is the total number of noise trials. The PSNR [46] measures the ratio between the maximum possible peak of the signal and the noise. The SSIM [46,47] measures the similarity between the reconstructed image and the phantom. The VIF [48] evaluates the image quality based on the natural scene statistics and the image notion extracted by the human visual system. The MAE [49] calculates the mean absolute error between the reconstructed image and the phantom. In Table 1, the best results, obtained from the SDPL-LN method using the SD roughness measure, are highlighted in bold.

**Figure 7:**
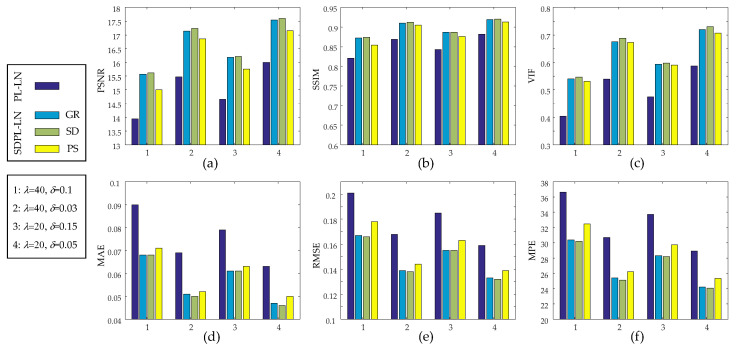
Performance comparison of PL-LN and SDPL-LN in terms of six image quality assessments: (**a**) PSNR; (**b**) SSIM; (**c**) VIF; (**d**) MAE; (**e**) RMSE; (**f**) MPE.

Figure 7 visualizes the quantitative results for the six IQAs presented in Table 1 through bar graphs, with each IQA depicted individually. The abscissa indexes the group number (1 to 4) for parameter settings (two distinct levels of initial parameter values for both *λ* and *δ*) used in Table 1. It is evident that the SDPL-LN methods clearly outperform the PL-LN method in all IQAs.

**Figure 8:**
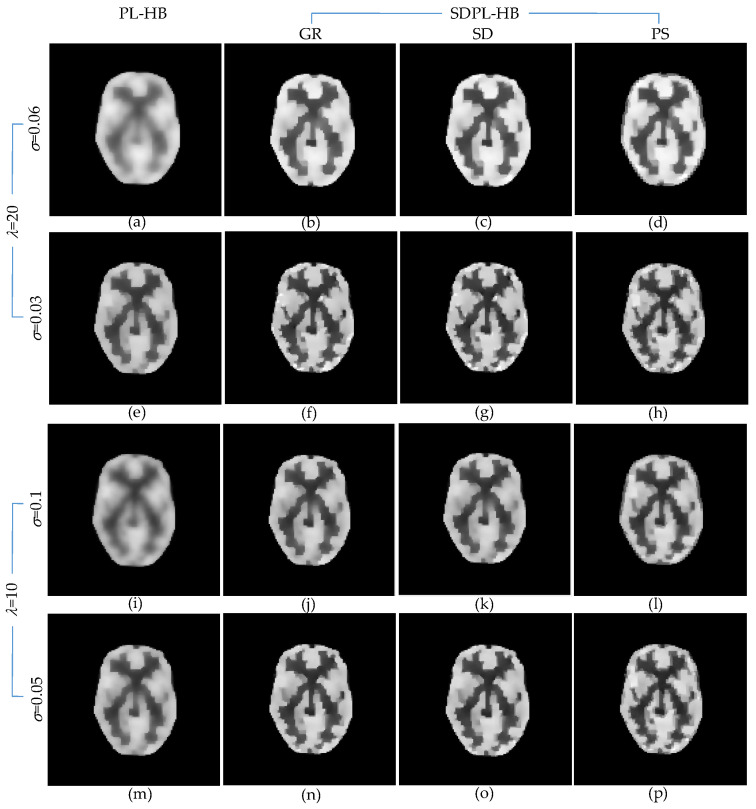
Anecdotal reconstructions using PL-HB and SDPL-HB with two different (high and low) levels of *λ* and two different (high and low) levels of *σ* for each *λ*. (The results in the first column (**a**,**e**,**i**,**m**) are PL-HB reconstructions, whereas the rest of the results are SDPL-HB reconstructions). (**a**–**h**) *λ* =20 (**i**–**p**) *λ* =10 (**a**–**d**) *σ* = 0.06; (**e**–**h**) *σ* = 0.03; (**i**–**l**) *σ* = 0.1; (**m**–**p**) *σ* = 0.05.

Figure 8 presents the anecdotal reconstructions using the HB penalty function, following the same layout as Figure 6 for the LN penalty function. Similar to the findings in Figure 6, the SDPL reconstructions consistently exhibit superior preservation of details compared to the standard PL reconstructions across all hyperparameter settings.

**Figure 9:**
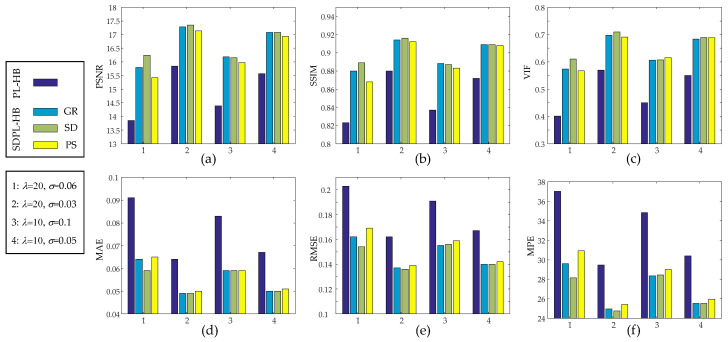
Performance comparison of PL-HB and SDPL-HB in terms of six different image quality assessments: (**a**) PSNR; (**b**) SSIM; (**c**) VIF; (**d**) MAE; (**e**) RMSE; (**f**) MPE.

Table 2 presents a performance comparison between the PL-HB and SDPL-HB methods based on six different IQAs. Again, the SDPL methods demonstrate the best outcomes. Although the best results are distributed across three different roughness measures, the differences among them are practically negligible. Figure 9 presents bar graphs visualizing the quantitative results in Table 2. The results clearly demonstrate that the SDPL-HB methods outperform the PL-HB method across all IQAs.

**Figure 10:**
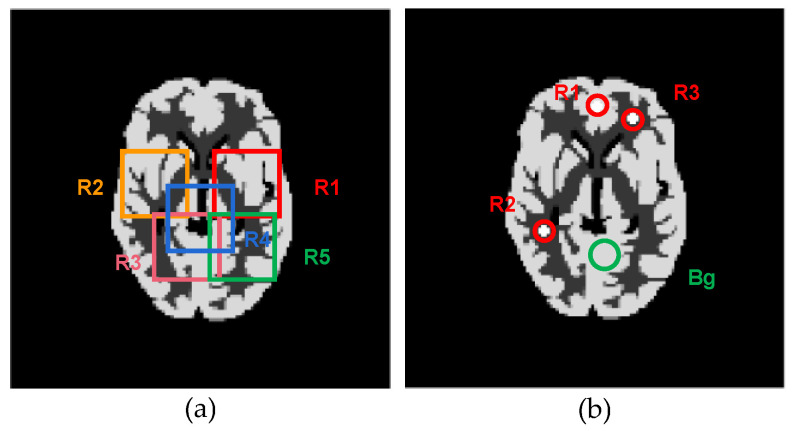
ROIs superimposed on the phantom image: (**a**) ROIs for regional percentage error; (**b**) ROIs for contrast recovery coefficient.

**Figure 11:**
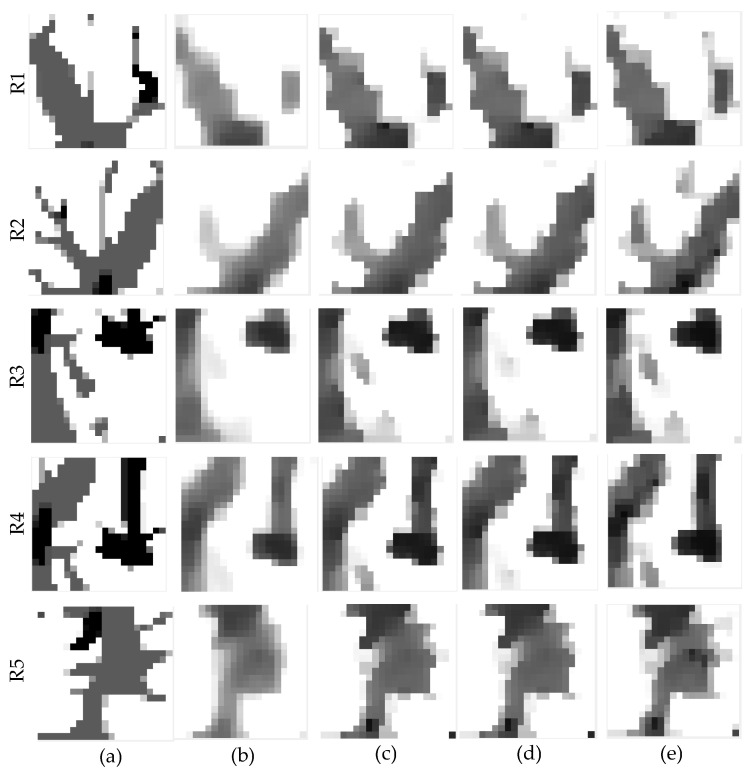
Zoomed-in images of PL-LN and SDPL-LN reconstructions using ROIs in Figure 8a. (**a**) phantom; (**b**) PL-LN; (**c**) SDPL-LN-GR; (**d**) SDPL-LN-SD; (**e**) SDPL-LN-PS.

**Figure 12:**
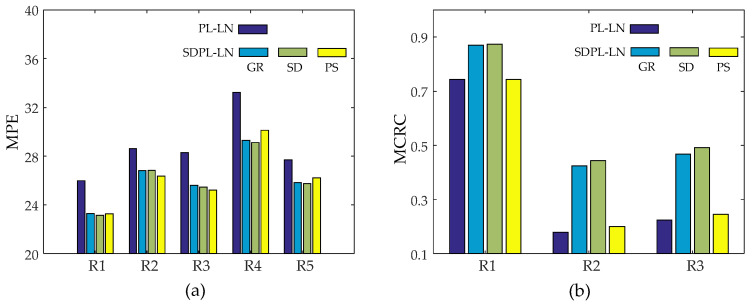
Regional performance comparison between the PL-LN and SDPL-LN methods: (**a**) regional mean percentage error (MPE) for ROIs shown in Figure 10a; (**b**) mean contrast recovery coefficient (MCRC) for ROIs shown in Figure 10b.

To evaluate the regional performance of our method, we first selected regions of interest (ROIs) as shown in Figure 10, and performed regional studies using the PL-LN and SDPL-LN reconstructions obtained with the same initial values of *λ* and *δ*, respectively. Figure 11 shows the five zoomed-in rectangular regions R1-R5 in Figure 10a, where the images in Figure 11a are zoomed-in regions of the phantom, Figure 11b zoomed-in regions of PL-LN reconstructions, Figure 11c zoomed-in regions of SDPL-LN-GR reconstructions (with the GR roughness measure), Figure 11d zoomed-in regions of SDPL-LN-SD, and Figure 11e zoomed-in regions of SDPL-LN-PS. As already seen in Figure 6, the SPDL-based methods clearly outperform the standard PL method, which is also verified in terms of the regional MPEs represented by the bar graphs shown in Figure 12a.

Figure 10b shows the three circular ROIs and one circular background region used for calculating the contrast recovery coefficient (CRC). The CRC is a metric that evaluates how well the algorithm restores the contrast of an ROI with respect to its background. The regional CRC is defined as

(18) $$CRC_{R}=CR_{R}/CR_{R0},$$

where ${\mathrm{CR}}_{R}=\left|{\hat{A}}_{R}-{\hat{A}}_{Bg}\right|/{\hat{A}}_{Bg}$, ${\hat{A}}_{R}=\left(1/T\right)\sum_{j\in R}{\hat{f}}_{j}$ denotes the mean activity in each ROI, ${\hat{A}}_{Bg}$ is the mean activity in the background region, and ${\mathrm{CR}}_{R0}$ is the true contrast in the phantom. Note that the value of CRC indicates the performance of the algorithm, with higher values closer to one indicating better performance.

Figure 12b presents the regional mean CRC (MCRC) calculated over *K* = 50 independent noise trials, which is defined as

(19) $$MCRC_{R}=\frac{1}{K}\sum_{k=1}^{K}CRC_{R}^{k},$$

where $CRC_{R}^{k}$ stands for the regional CRC calculated from the *k*-th noise realization. It is evident that the SDPL-based methods with GR and SD roughness measures remarkably outperform the standard PL method in terms of the MCRC in all three ROIs.

### 3.2. Qualitative Validation Using Physically Acquired Data

**Figure 13:**
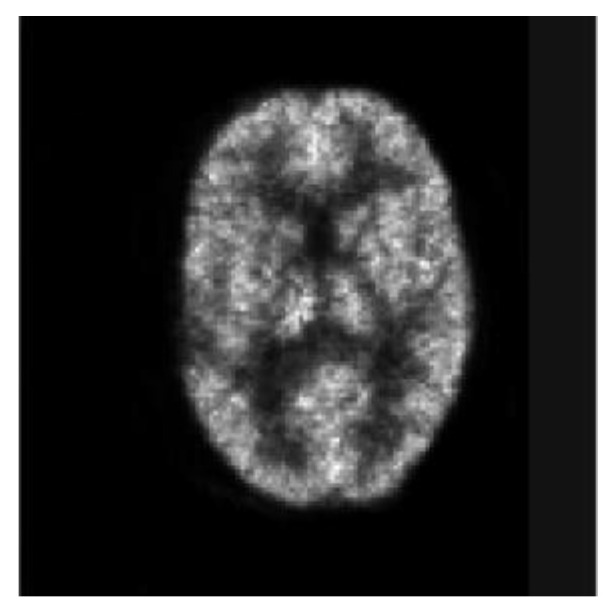
EM-ML reconstruction (40 iterations) from physically acquired projection data.

To observe qualitatively the efficacies of our SDPL methods, we acquired physical data using a GE Advance PET scanner, which contains 18 detector rings yielding 35 slices at 4.25 mm center-to-center slice separation. We acquired 2D data from the physical Hoffman brain phantom using the scanner’s high sensitivity mode with septa in. The sinogram dimension was 145 detector pairs and 168 angles. The projection data were acquired for 10 min from an ^18^FDG scan. The corresponding number of detected coincident counts was approximately 1,000,000. Figure 13 shows the typical noise level observed in the EM-ML reconstruction with 40 iterations, obtained from the physical PET data. Since there is no ground-truth data available for this experiment, the efficacies of using the COSEM-PL and COSEM-SPDL methods can be observed qualitatively by comparing their results with the EM-ML reconstruction. It is important to note that, compared to the reconstructions shown in Figure 6 and Figure 8, which were obtained from the digital phantom, the resolution of the EM-ML reconstruction for the real PET data is significantly low, which may limit our qualitative observation of the efficacies of using the SPDL methods in the real data experiments.

**Figure 14:**
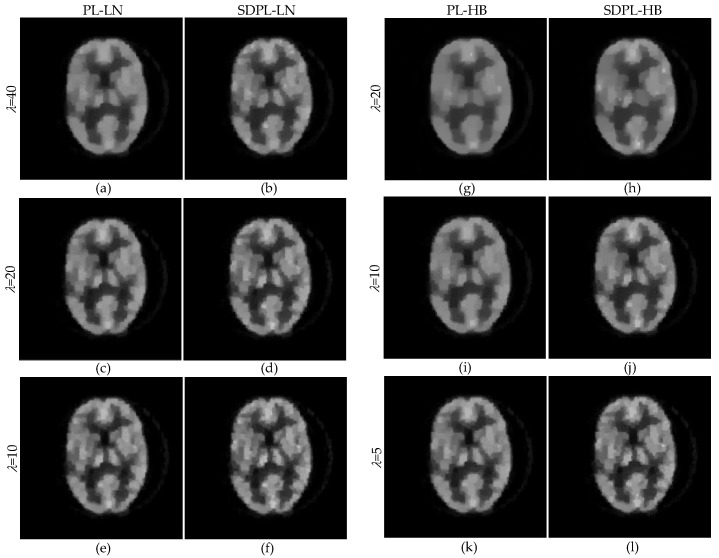
COSEM-PL reconstructions from physically acquired data: (**a**) PL-LN with *λ* = 40; (**b**) SDPL-LN with *λ* = 40; (**c**) PL-LN with *λ* = 20; (**d**) SDPL-LN with *λ* = 20; (**e**) PL-LN with *λ* = 10; (**f**) SDPL-LN with *λ* = 10; (**g**) PL-HB with *λ* = 20; (**h**) SDPL-HB with *λ* = 20; (**i**) PL-HB with *λ* = 10; (**j**) SDPL-HB with *λ* = 10; (**k**) PL-HB with *λ* = 5; (**l**) SDPL-HB with *λ* = 5.

Figure 14 shows two groups of images, Figure 14a–f and Figure 14g–l, reconstructed by COSEM-PL with the LN and HB penalty functions, respectively. For the LN penalty, the smoothing parameter values were set to 40, 20, and 10 for Figure 14a,b, Figure 14c,d, and Figure 14e,f, respectively. For the HB penalty, the smoothing parameter values were set to 20, 10, and 5 for Figure 14g,h, Figure 14i,j, and Figure 14k,l, respectively. For each value of *λ*, a value of *δ* (or *σ*) was chosen for the standard PL first, and it was used as an initial value of *δ* (or *σ*) for the SDPL. For each value of *λ*, a close inspection reveals that, as already observed in Figure 6 and Figure 8 using the digital phantom, the SDPL method further improves the reconstruction of fine details. In fact, the visual improvement from the standard PL reconstruction to the SDPL reconstruction in Figure 14 is not as stunning as that in Figure 6 and Figure 8. This is presumably due to the fact that the physical factors that affect the quality of reconstruction were not modeled in our reconstruction algorithms. While the attenuation correction was done by a conventional method that uses the ratio of the measurements in the blank and transmission scans, the factors to model scattered and random coincidences were not included in our reconstruction algorithms. In this case, the measurement is not strictly Poisson. (Our future work includes modeling the physical factors in the likelihood term and expanding accordingly the overall energy function described in (11)).

## 4. Summary and Conclusions

We have presented similarity-driven hyperparameter fine-tuning methods for penalized-likelihood image reconstruction in PET. Our proposed method aims to optimize the regularization parameter by leveraging similarity information between neighboring patches, leading to improved image quality and quantitative accuracy.

The experimental results obtained from the digital phantom studies demonstrated the effectiveness of the proposed method in achieving superior image reconstruction performance compared to the conventional PL method with fixed hyperparameters. By incorporating similarity information into the hyperparameter optimization process, the proposed method effectively balanced the trade-off between noise reduction and preservation of fine details, resulting in visually enhanced images with reduced noise. Our numerical studies supported the visual comparison by showing better quantitative performance of the proposed method across multiple image quality metrics. Finally, the additional results from the physical experiments using the real PET data also supported the good performance of the proposed method. However, to fully evaluate the clinical potential and generalizability of the proposed method, more comprehensive investigations that incorporate the physical factors, such as attenuation, scattered, and random coincidences, into reconstruction algorithms for real PET scans, are needed.

We acknowledge here that, besides the regularization approach employing CNQ penalties discussed in this study, there exist several other types of regularization methods used in PET reconstruction, which encompass total variation regularization [50,51,52], sparse coding-based regularization [53,54,55], and low-rank/sparse decomposition-based regularization [56]. These regularization methods also involve hyperparameters that significantly impact the quality of the reconstructed image. Since our proposed method specifically focuses on CNQ penalties, further investigation is required to determine the feasibility of integrating it with these diverse regularization methods.

We also note that, as the proposed method requires initially tuned hyperparameters for the entire image, it is not fully automated. For our future work, we would continue to seek a more advanced approach to optimizing the regularization parameter to fully automate the tuning process. One possible approach may be to use our method in conjunction with machine learning-based parameter tuning methods [21,22,23] so that the parameters initially tuned by machine learning for the entire image can be refined by our method for further improvements in reconstruction accuracy. However, we acknowledge the inherent challenge for machine learning methods to incorporate the additional control parameters responsible for adjusting edge reservation sensitivity by modifying the shape of the penalty function. Despite this challenge, we expect that our proposed method, in conjunction with more advanced machine learning-based approaches that can handle the control parameters, will substantially reduce the dependence on subjective trial-and-error hyperparameter tuning in regularized PET reconstruction.

## Figures and Tables

**Table 1:** Quantitative performance comparison of PL-LN and SDPL-LN.

	IQA Metrics	PL-LN	SDPL-LN		
			GR	SD	PS
λ = 40 δ = 0.1	PSNR(dB)	13.943	15.569	**15.621**	14.999
	SSIM	0.821	0.872	**0.874**	0.854
	VIF	0.404	0.540	**0.547**	0.531
	MAE	0.090	**0.068**	**0.068**	0.071
	RMSE	0.201	0.167	**0.166**	0.178
	MPE	36.651	30.396	**30.216**	32.459
λ = 40 δ = 0.03	PSNR(dB)	15.475	17.133	**17.232**	16.856
	SSIM	0.869	0.910	**0.912**	0.905
	VIF	0.539	0.675	**0.688**	0.672
	MAE	0.069	0.051	**0.050**	0.052
	RMSE	0.168	0.139	**0.138**	0.144
	MPE	30.728	25.387	**25.101**	26.210
λ = 20 δ = 0.15	PSNR(dB)	14.659	16.186	**16.222**	15.756
	SSIM	0.843	**0.887**	**0.887**	0.876
	VIF	0.474	0.593	**0.598**	0.590
	MAE	0.079	**0.061**	**0.061**	0.063
	RMSE	0.185	**0.155**	**0.155**	0.163
	MPE	33.754	28.311	**28.194**	29.748
λ = 20 δ = 0.05	PSNR(dB)	16.001	17.543	**17.601**	17.151
	SSIM	0.882	0.919	**0.920**	0.913
	VIF	0.587	0.720	**0.730**	0.706
	MAE	0.063	0.047	**0.046**	0.050
	RMSE	0.159	0.133	**0.132**	0.139
	MPE	28.922	24.217	**24.056**	25.335

**Table 2:** Quantitative performance comparison of PL-HB and SDPL-HB.

	IQA Metrics	PL-HB	SDPL-HB		
			GR	SD	PS
*λ* = 20 *σ* = 0.06	PSNR(dB)	13.857	15.798	**16.239**	15.421
	MSSIM	0.823	0.880	**0.889**	0.868
	VIF	0.401	0.574	**0.611**	0.567
	MAE	0.091	0.064	**0.059**	0.065
	RMSE	0.203	0.162	**0.154**	0.169
	MPE	37.018	29.605	**28.142**	30.920
*λ* = 20 *σ* = 0.03	PSNR(dB)	15.838	17.277	**17.353**	17.134
	SSIM	0.880	0.914	**0.916**	0.912
	VIF	0.569	0.697	**0.710**	0.691
	MAE	0.064	**0.049**	**0.049**	0.050
	RMSE	0.162	0.137	**0.136**	0.139
	MPE	29.469	24.971	**24.755**	25.386
*λ* = 10 *σ* = 0.1	PSNR(dB)	14.387	**16.176**	16.149	15.974
	SSIM	0.837	**0.888**	0.887	0.883
	VIF	0.450	0.606	0.607	**0.615**
	MAE	0.083	**0.059**	**0.059**	**0.059**
	RMSE	0.191	**0.155**	0.156	0.159
	MPE	34.826	**28.346**	28.434	29.011
*λ* = 10 *σ* = 0.05	PSNR(dB)	15.564	**17.086**	**17.086**	16.938
	SSIM	0.872	**0.909**	**0.909**	0.908
	VIF	0.550	0.684	**0.689**	0.688
	MAE	0.067	**0.050**	**0.050**	0.051
	RMSE	0.167	**0.140**	**0.140**	0.142
	MPE	30.412	**25.526**	25.527	25.963

## Data Availability

The data presented in this study are available upon request. Please contact the corresponding author.
